# An Efficient Computational Method for Calculating Ligand Binding Affinities

**DOI:** 10.1371/journal.pone.0042846

**Published:** 2012-08-20

**Authors:** Atsushi Suenaga, Noriaki Okimoto, Yoshinori Hirano, Kazuhiko Fukui

**Affiliations:** 1 Computational Biology Research Center, National Institute of Advanced Industrial Science and Technology, Koto-ku, Tokyo, Japan; 2 Computational Biology Research Core, Quantitative Biology Center, RIKEN, Kobe, Hyogo, Japan; UMR-S665, INSERM, Université Paris Diderot, INTS, France

## Abstract

Virtual compound screening using molecular docking is widely used in the discovery of new lead compounds for drug design. However, the docking scores are not sufficiently precise to represent the protein-ligand binding affinity. Here, we developed an efficient computational method for calculating protein-ligand binding affinity, which is based on molecular mechanics generalized Born/surface area (MM-GBSA) calculations and Jarzynski identity. Jarzynski identity is an exact relation between free energy differences and the work done through non-equilibrium process, and MM-GBSA is a semimacroscopic approach to calculate the potential energy. To calculate the work distribution when a ligand is pulled out of its binding site, multiple protein-ligand conformations are randomly generated as an alternative to performing an explicit single-molecule pulling simulation. We assessed the new method, multiple random conformation/MM-GBSA (MRC-MMGBSA), by evaluating ligand-binding affinities (scores) for four target proteins, and comparing these scores with experimental data. The calculated scores were qualitatively in good agreement with the experimental binding affinities, and the optimal docking structure could be determined by ranking the scores of the multiple docking poses obtained by the molecular docking process. Furthermore, the scores showed a strong linear response to experimental binding free energies, so that the free energy difference of the ligand binding (ΔΔG) could be calculated by linear scaling of the scores. The error of calculated ΔΔG was within ≈±1.5 kcal•mol^−1^ of the experimental values. Particularly, in the case of flexible target proteins, the MRC-MMGBSA scores were more effective in ranking ligands than those generated by the MM-GBSA method using a single protein-ligand conformation. The results suggest that, owing to its lower computational costs and greater accuracy, the MRC-MMGBSA offers efficient means to rank the ligands, in the post-docking process, according to their binding affinities, and to compare these directly with the experimental values.

## Introduction

Most drugs are small chemical compounds. However the molecular mechanisms of action of a lot of drugs are not known. Because protein-protein and protein-ligand interactions play a crucial role in biological functions and reactions, such as enzyme catalysis and intracellular signal transduction, recently drugs which bind to a target protein and then inhibit protein-protein interaction or enzyme reactions have become of a subject of interest. The candidates of these drugs should strongly and specifically bind to the target proteins, thus the accurate prediction of the binding affinity of a ligand for a protein is a critical element in drug discovery. Because drugs have traditionally been discovered through trial and error, often involving a great deal of expense and time, computer-aided drug design has become important, owing to its comparative facility and lower cost. With recent advancements in computer technology and methodology, powerful parallel computers are now available for use in increasingly efficient computer-aided drug design. However, computationally accurate prediction of protein-ligand binding affinity remains a great challenge.

One of the most popular approaches to computer-aided drug design is the molecular docking method, involving the computational screening and ranking of a library of ligands to identify potential lead chemical compound candidates. Many docking programs [1]–[8] involve two operations: docking and scoring. In scoring, the binding affinity of a ligand for a target protein is calculated by using an approximated scoring function, based on a simplified empirical force field or potential of mean force, for the sake of computational speed. Numerous studies using docking programs have shown that these screenings have a higher enrichment of active compounds than random screening [9], [10]; however, they suffer from false positives and false negatives, and are not sufficiently accurate to rank compounds according to their binding affinities [11]. As a consequence, the docking results must be post-processed with more accurate methods for calculating the binding affinities (scores), before the ranking and selection of potential lead compounds. Especially, in the case that no experimental screening is available or only very few compounds have to be selected, more precisely method to rank the ligands according to their binding affinities should be needed.

All-atom molecular dynamics (MD) simulation with explicit solvent, in combination with efficient and rigorous free energy calculation methods, can accurately predict the binding free energy of ligands to proteins [12]. These free energy calculation methods include the free energy perturbation method, thermodynamic integration, umbrella sampling, the potential of mean force method, the double-annihilation method, the double-decoupling method, and single-molecule pulling simulations. Numerous studies using these methods have reported that calculated binding free energies are quantitatively in excellent agreement with experimental values [13]–[16]; however, the respective methods are computationally too expensive to be employed in the post-docking process and also too difficult to apply to a wide variety of chemical compounds [17].

Alternatives to these rigorous free energy calculations are offered by linear response approximation (LRA) [18] and the linear interaction energy (LIE) [19], where only the ligand-bound and unbound states are simulated. Combining the semimacroscopic approach based on protein dipoles Langevin dipoles (PDLD/S) and LRA (PDLD/S-LRA) reduces the computational cost without loss of accuracy [20], [21]. Another widely used semimacroscopic approach is the molecular mechanics-Poisson Boltzmann (or Generalized Born) surface area (MM-PB(GB)SA) method [22], [23]. Because the free energy is thermodynamically statistical, energies should be averaged over the MD trajectory in these methods. However, MD simulations for the post-docking process, especially those in an explicit solvent, are time-consuming. Although free energy calculations using the MM-GB(PB)SA method are usually made on an ensemble of structures sampled during MD simulation in explicit solvent, using a single energy-minimized structure to MM-GB(PB)SA method often showed reasonable approximation for rapidly estimating the ligand binding free energies (S-MMGB(PB)SA) [24]–[26]. The ligand binding free energies can be easily and rapidly calculated by the S-MMGB(PB)SA method; however, it is difficult to be employed in most proteins, especially flexible proteins, because of its too rough approximation based on the use of a single conformation. In terms of theoretical foundations, computational costs, and effectiveness in calculating absolute binding free energies, either the LIE or the PDLD/S-LRA approach would provide an efficient method after potentially lead candidates have been narrowed down to several tens of compounds [20], [21]. Thus, some other method is required, to act as a bridge between the molecular docking method and the LIE, PDLD/S-LRA, or other more rigorous methods, in order to efficiently, and with low computational cost, enrich the small number of potential candidates from the large chemical compound library.

**Figure 1:**
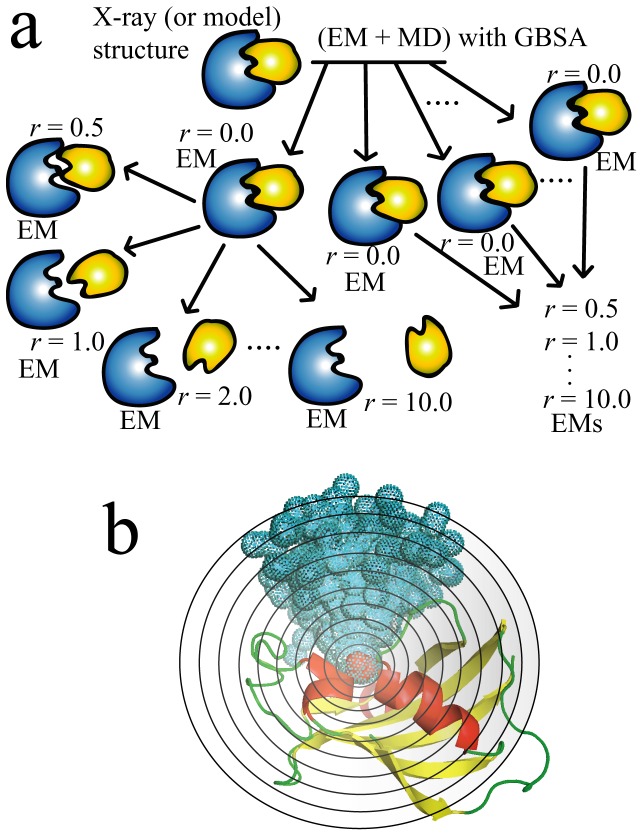
Schematic representation of the MRC-MMGBSA method. (**a**) In the MRC-MMGBSA procedure, ligand molecules are randomly generated around a target protein with varying protein-ligand distance *r* and orientation. EM indicates the energy-minimization. (**b**) A 3D illustration of the distribution of ligand molecules randomly generated around the target protein, where the target protein is drawn by ribbon representation, and the colored spheres represent the centers of mass of the respective ligands.

In this study, we describe an efficient method for calculating the protein-ligand binding affinities, namely the MRC-MMGBSA (Multiple Random Conformation-MMGBSA) method, which is based on the MM-GBSA calculation and Jarzynski identity [27], [28]. Jarzynski identity is an exact relation between free energy differences and the work done through non-equilibrium processes. To calculate the work done when the ligand is pulled out of the binding site, single-molecule pulling MD simulation which is mimicking the single molecule experiment is suitable [16], [29]–[31]. However, the single-molecule pulling MD simulation is computationally too expensive to be applied for large datasets of ligands which is including several millions compounds. In our method, the multiple protein-ligand conformations, whose ligands are rotatable with protein-ligand distance *r*, are randomly generated for statistical analysis, rather than performing single-molecule pulling MD simulations. The protein-ligand distance *r* is defined as the difference in the distance between the respective centers of mass of a given ligand in the original (X-ray, NMR or model structure) configuration, and in a related randomly generated position. In this study, *r* was set at 0.0, 0.5, 1.0, 2.0, 3.0…10.0, and a hundred conformations for each distance *r* were generated. In this process, six random numbers were generated, *x*, *y*, and *z* of rotation angles (*θ_x_*, *θ_y_*, *θ_z_*) and protein-ligand distance *r* (*r_x_*, *r_y_*, and *r_z_*) [wherein *r* ^2^ = (*r_x_* ^2^+*r_y_* ^2^+*r_z_* ^2^)]. These randomly generated conformations, with various values for the protein-ligand distance *r* and various ligand orientations, were subjected to energy minimization in implicit solvent with GBSA method, to calculate the potential energies. A schematic representation of the method is shown in Figure 1. The work when ligand is pulled out of its binding site can be approximately estimated from the potential energies (see method).

The MRC-MMGBSA method was assessed by evaluating the ligand binding affinities for four target systems: the FK506 binding protein (FKBP), bovine trypsin, the dipeptide binding protein (DPPA), and cyclin-dependent kinase 2 (CDK2). The computational results obtained from X-ray and modeled protein-ligand complex structures were found to be in good qualitative agreement with experimental binding affinities. This result indicates the higher ranking accuracy of the MRC-MMGBSA method. Next, to assess the ability for determining the optimal docking structure, which is similar to experimental protein-ligand complex structure, from among the multiple docking poses, the docking structures were built by AutoDock Vina [8], and the docking poses were rescored, using the MRC-MMGBSA method. By utilizing the calculated binding affinities (MRC-MMGBSA scores), the optimal docking structures could be determined from among the multiple docking poses. Importantly, the free energy difference of the ligand binding (ΔΔ*G*) could be obtained by linear scaling the scores within ≈±1.5 kcal•mol^−1^ of the experimental values. Overall, we conclude that the MRC-MMGBSA approach is an efficient method for use in the post-docking process, prior to the second screening process employing more rigorous methods such as the LIE or PDLS/S-LRA calculation, because of its low computational costs and higher ranking accuracy, especially in the case of flexible target proteins.

## Results

### Calculation of MRC-MMGBSA scores

We assessed the MRC-MMGBSA method by evaluating MRC-MMGBSA scores for four target systems (FKBP (10 ligands), trypsin (7 ligands), DPPA (7 ligands), and CDK2 (7 ligands)), and comparing these scores with experimental binding affinities [32]–[37]. The four systems included a variety of sizes and net charges for receptor proteins and ligands (e.g. FKBP = small sized, 107 amino acids (aa); trypsin and CDK2 = middle sized, 223 and 297 aa, respectively; and DPPA = large sized, 507 aa). The structures and net charges of the relevant ligands are summarized in the supporting information (SI), Figures S1, S2, S3, and S4 and Tables S1, S2, S3, and S4. In cases where the X-ray or NMR structure for the protein-ligand complex was not available, the protein-ligand complex structure was modeled (Tables S1, S2, S3, and S4).

**Figure 2:**
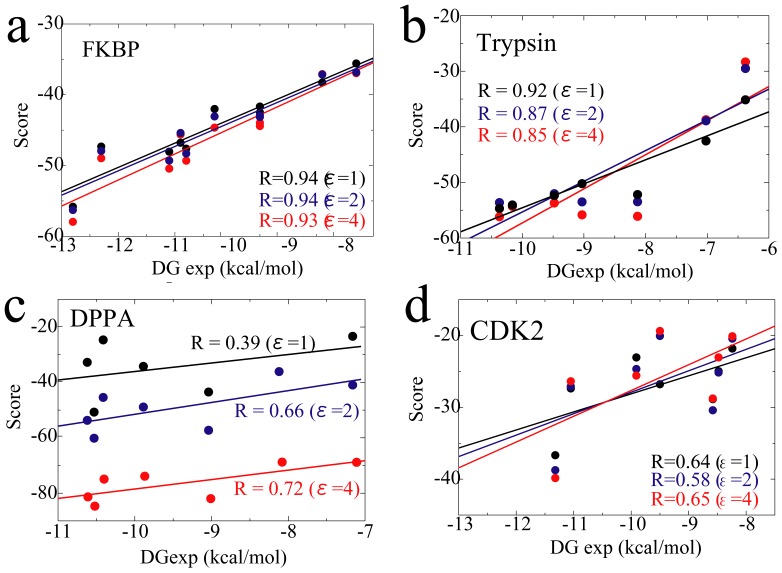
Correlation between MRC-MMGBSA scores and experimental binding affinities. The respective correlation coefficients (*R* values) are shown in the figures.

Figure 2 shows the correlation between the MRC-MMGBSA scores and experimental binding affinities. The scores were in good qualitative agreement with the experimental binding affinities. In the case of the FKBP, trypsin and CDK2 systems, the results showed no dependency on the dielectric constant *ε*, with high correlation coefficients *R*. In the case of the DPPA system, agreement depended entirely upon the *ε* value. It has been noted that a high dielectric constant is useful in achieving reorganization and polarization effects in highly charged systems [38]–[40]. For the FKBP system, the net charge of all ligands and the receptor protein were neutral (Table S1) and +4.0, respectively. For trypsin, the net charge of the ligands and receptor were neutral (one ligand) or +1.0 (six ligands) (Table S2) and +6.0, respectively. For CDK2, the net charge of the ligands and receptor were neutral (Table S4) and +9.0, respectively. Due to the *ε* dependency of the DPPA system, the dipeptides had high partial charges, with an N-terminal charge of +1.0, a C-terminal charge of −1.0, and/or a side-chain charge of −1.0 for Asp and +1.0 for Lys; although the net charge of the dipeptides used in this study were −1.0 (one ligand), 0.0 (five ligands), or +1.0 (one ligand) (Table S3), and the net charge of the receptor was also negatively large, −8.0.

The reason for the lower accuracy in the case of DPPA, in comparison to those of FKBP and trypsin, is that DPPA is the most difficult target among the three target proteins, because it shows conformational change upon ligand binding [41], [42]. The ligand (peptide) binding pocket of DPPA is open and accessible in the ligand-free state. When ligand is bound, the ligand binding pocket is closed and the ligand is buried by hinge motion of DPPA. Thus, in the case of DPPA, we used the holo-type (closed state) receptor only for the protein-ligand distance *r* = 0.0 (ligand-bound state), and the apo-type (open state) receptor for all other protein-ligand distances. The holo-type DPPA receptor can be used only for the protein-ligand distance *r* = 0.0, because the ligand molecule crashes into the receptor, especially at small *r* values. Thus we did not try the case where only the holo-type receptor is used for all *r* in the DPPA. When using only the apo-type DPPA receptor for all protein-ligand distances, we obtained consistently poor results (*R* = −0.46 for *ε* = 1, *R* = −0.23 for *ε* = 2, and *R* = −0.04 for *ε* = 4). Therefore, we concluded that both apo- and holo-type receptors should be utilized when calculating the ligand binding affinities in the case of flexible proteins, and that the MRC-MMGBSA method can correctly rank the ligands according to the scores, not only for rigid target proteins but also for flexible ones.

In the case of CDK2, the difficulty (low accuracy) may be caused by two reasons. One is that large hydrophobic region covers the surface of the ligand binding pocket. The hydrophobic interaction is difficult to reproduce by the implicit solvent GBSA method. The other is the flexibility (adjustable the shape) of the binding pocket. From comparison among several crystal structures of CDK2-ligand complex, the shapes (volumes) of the ligand binding pocket ware different for each ligand. MRC-MMGBSA method made up for the above two shortcomings (correlation coefficient *R* between experimental and calculated binding affinities was 0.65 for *ε* = 4.0, Table 1) by calculating the energies for multiple conformations.

**Table 1:** Correlation coefficients *R* between the experimental and calculated binding affinities.

Target proteins	S-MMPBSA			S-MMGBSA			MRC-MMGBSA		
	*ε* = 1	*ε* = 2	*ε* = 4	*ε* = 1	*ε* = 2	*ε* = 4	*ε* = 1	*ε* = 2	*ε* = 4
FKBP	0.74	0.82	0.84	0.82	0.90	0.92	0.94	0.94	0.93
Trypsin	0.42	0.88	0.88	0.84	0.92	0.90	0.92	0.87	0.85
DPPA	NC*	NC	NC	0.49†	0.52†	0.53†	0.39	0.66	0.72
CDK2	−0.14	−0.09	0.05	0.16	0.34	0.20	0.64	0.58	0.65

* Not calculated.

† For calculation of the free energy of the receptor, the apo-type structure was used.

The S-MMGB(PB)SA approach is well known for its simplicity as an efficient and widely used method for calculating ligand binding affinities. Therefore, we next compared the results obtained from the MRC-MMGBSA and S-MMGB(PB)SA method, and found that the S-MMGBSA produced better results than the S-MMPBSA (Table 1). The correlation coefficients obtained from the S-MMGBSA were as high as those obtained by the MRC-MMGBSA calculation, except in the cases of DPPA and CDK2 (Table 1). In this study, all the protein-ligand complex structures were X-ray or model structures, indicating that all the protein-ligand binding modes were correct (or nearly correct); hence the good results obtained by the S-MMGBSA calculation. On the other hand, in the cases of DPPA and CDK2, only the MRC-MMGBSA scores showed good correlation with experimental values (*ε* = 4). This suggests that flexible proteins (e.g. DPPA) and kinase (e.g. CDK2) may be problematic for the S-MMGB(PB)SA calculation. However, on the whole, the MRC-MMGBSA score correctly ranked the ligands in accordance with the experimental binding affinities, with a correlation coefficient *R* greater than 0.65, even if the target protein underwent large conformational change, and the large hydrophobic regions are located in the ligand binding pocket (the hydrophobic interactions are dominant for the protein-ligand interactions), and the shape of the ligand binding pocket is adjustable, such as CDK2, which are typically difficult cases for computational screening.

### Parameters for the MRC-MMGBSA calculation

In the MRC-MMGBSA calculation, three parameters must be determined: first, the number of energy-minimization steps (*N*min); second, the number of conformations randomly generated for each protein-ligand distance *r* (*N*conf); and third, the increments and maximum of the protein-ligand distance *r*. For calculation of the scores, a parameter set of *N*conf = 100, *N*min = 100, *r* = {0.0, 0.5, 1.0, 2.0, 3.0, 4.0, 5.0, 6.0, 7.0, 8.0, 9.0, 10.0} (12 points) was used as the default. In total, 100×12 = 1,200 conformations were generated.

**Figure 3:**
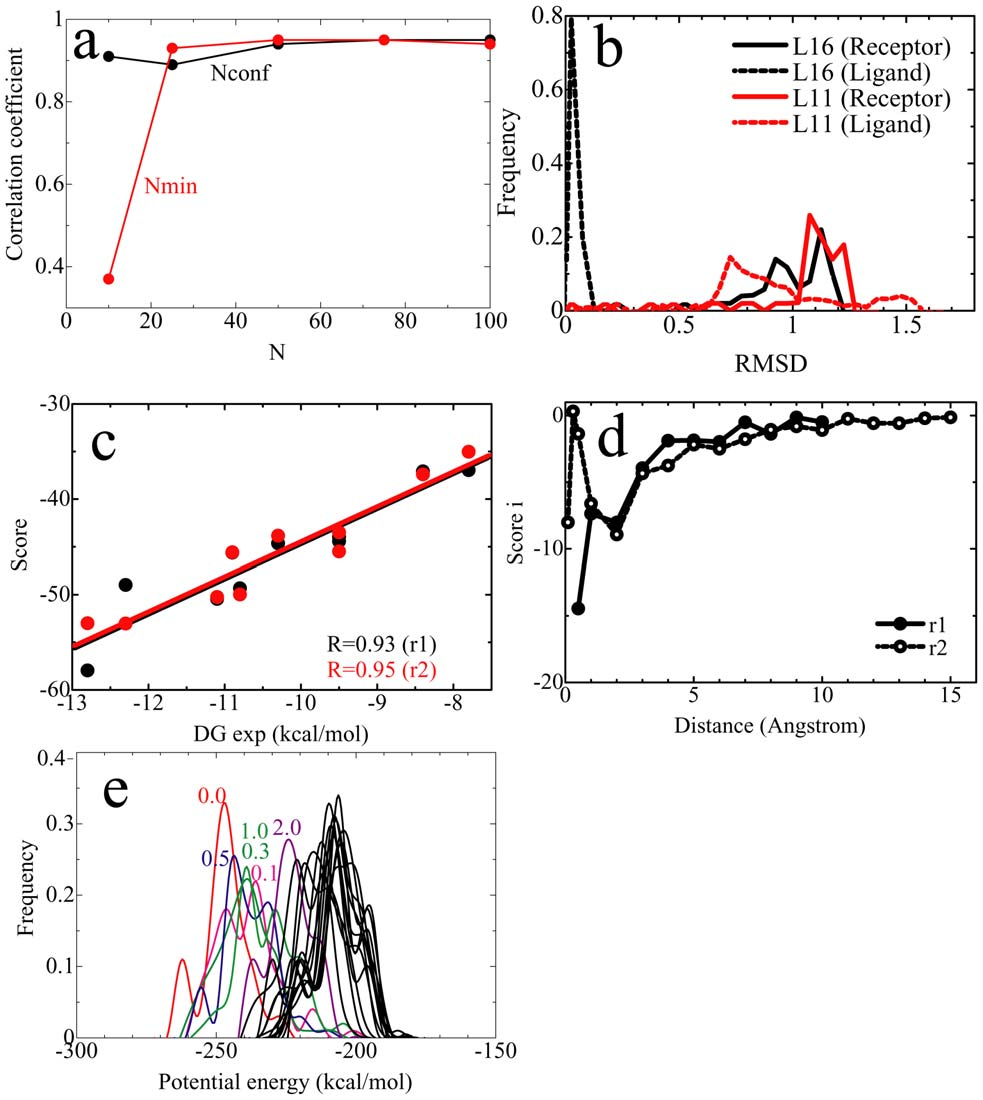
Typical results of parameter set assessment. (**a**) Plots of correlation coefficients between experimental and calculated binding affinities versus *N*conf (black) and *N*min (red). In the *N*conf assessment, *N*min = 100 was used. In the *N*min assessment, *N*conf = 100 was used. (**b**) The structural diversity of receptor proteins (trypsin) and ligands. The cases involving the smallest (L16) and the largest (L11) ligands are shown. The distribution of root mean square displacement (RMSD) of the respective ligands, between the reference (X-ray, NMR or model) and randomly generated conformations, by fitting the receptor protein, is plotted. (**c**) The correlation between MRC-MMGBSA scores and experimental binding affinities, under *r*1 and *r*2, for FKBP. (**d**) The correlation between the MRC-MMGBSA score*_i_* and the protein-ligand distance *r* of L04, for FKBP. (**e**) The potential energy distribution of each protein-ligand distance *r* of the largest ligand L04, for FKBP.

To assess the validity of the default parameter set, we calculated the scores using several different parameter sets, and compared the correlation coefficients between the scores and the experimental values. From the result, we found that stable results were obtained with *N*conf>50 and *N*min>50 (Figure 3A). Although *N*conf appeared to have only a small effect on the results, the larger *N*conf is needed in order to take into account entropic effects. In this study, the respective structures of receptor protein and ligand, which were obtained by short (about 10 ps) MD simulation sampling of the bound state (*r* = 0.0, 100 conformations) and energy-minimization of the randomly generated conformations (1,100 conformations), showed significant structural diversity (Figure 3B). The small ligands showed sharp diversity, while the large ligands showed large diversity in all systems. This suggests that structural diversity makes an entropic contribution to the MRC-MMGBSA calculation and might be a reason for the greater accuracy of the MRC-MMGBSA method than the S-MMGBSA method, although it is surely insufficient for sampling wide range of conformational space.

Next, we determined the effect of the increments and maximum protein-ligand distance *r* on the calculation results. We calculated the scores using both the default values of *r*1 = {0.0, 0.5, 1.0, 2.0, 3.0, 4.0, 5.0, 6.0, 7.0, 8.0, 9.0, 10.0} and those of *r*2 = {0.0, 0.1, 0.25, 0.5, 1.0, 2.0, 3.0, 4.0, 5.0, 6.0, 7.0, 8.0, 9.0, 10.0, 11.0, 12.0, 13.0, 14.0, 15.0}. The respective scores were very similar (Figure 3C). As further analysis, we focused on one of the score components, namely, the score for each distance *r* (*score_r = i_*, see Equation (3) in Methods below). This score*_r_* increased asymptotically and clearly approached zero beyond 10 Å in both the *r*1 and *r*2 calculations (Figure 3D), which supports the use of the maximum distance of *r* = 10.0 Å. The score*_r_* at *r* = 0.5 Å was different for the *r*1 and *r*2 calculations, while the score*_r_* beyond 1.0 Å was almost the same. This means that the total score*_r_* of *r* = 0.1, 0.25 and 0.5 in *r*2 is roughly equal to the score*_r_* at *r* = 0.5 in *r*1, as expected. Furthermore, the energy distributions of each distance *r* were sufficiently overlapped (Figure 3E). Overall, the results suggest that *r*1 is usable as a rough calculation of *r*2, allowing for a reduction in computational costs without the loss of accuracy. In summary, we concluded that the parameter set employed in this study is appropriate for the calculation, and offers a standard for the analysis of other proteins as well, since we confirmed its validity by using a variety of target proteins and ligands, varying in size and charge.

### Rescoring of docking poses

In the docking process, the top-scored docking pose does not always correspond to the optimal docking structure. Thus, the abilities to determine the optimal docking structure among multiple docking poses generated by the docking process, as well as to correctly rank the ligands according to their binding affinities, are important for successful computational screening. Here we built the protein-ligand docking structures of the FKBP, trypsin, DPPA, and CDK2 systems using the AutoDock Vina program [8], generating five docking poses for each ligand. These docking poses were then rescored using the MRC-MMGBSA and S-MMGBSA methods, in order to determine the optimal docking structure.

In this study, we defined the optimal docking structure as one that shows less than a 2.0 Å root mean square (RMS) displacement in the ligand position by fitting the receptor protein, between the reference (X-ray, NMR or model) and the actual the docking structures. For all the systems, nearly all the top-scored poses obtained by the MRC-MMGBSA and S-MMGBSA methods corresponded to the optimal protein-ligand complex structure (Tables S5, S6, S7, and S8). However, the MRC-MMGBSA approach showed a greater ability to rank the top-scored ligands according to their binding affinities than did the S-MMGBSA method (Table 2). For example, in the case of *ε* = 4, the correlation coefficients between the experimental and calculated binding affinities (scores) for the optimal protein-ligand complex structures determined by MRC-MMGBSA method were greater than those determined by S-MMGBSA method. These results indicate that the S-MMGBSA approach is an adequate method for determining the optimal protein-ligand complex from among the multiple docking poses and has the advantage of rapid calculation, whereas the MRC-MMGBSA is efficient in ranking the ligands after that optimal docking structure has been determined by the S-MMGBSA method. Finally, the use of a dielectric constant *ε* of 4 offered the best results with regard to ranking the ligands according to their binding affinities and determining the optimal protein-ligand complex structure among the multiple docking poses.

**Table 2:** Correlation coefficients *R* between the experimental and calculated binding affinities (scores) for the top-scored docking poses.

Target proteins	AutoDock Vina	S-MMGBSA			MRC-MMGBSA		
		*ε* = 1	*ε* = 2	*ε* = 4	*ε* = 1	*ε* = 2	*ε* = 4
FKBP	0.79	0.79	0.82	0.79	0.83	0.77	0.89
Trypsin	0.86	0.87	0.93	0.93	0.73	0.94	0.98
DPPA	−0.15	0.29*	0.54*	0.37*	0.36	0.69	0.77
CDK2	−0.20	−0.04	0.34	0.33	0.30	0.74	0.89

* For calculation of the free energy of the receptor, the apo-type structure was used.

### Direct comparison of MRC-MMGBSA scores with experimental binding affinities

If we assume that the MRC-MMGBSA scores are linearly related to the absolute binding free energies (Δ*G*), we can determine the weighting factor *α*. Here, the simplest approximation, Δ*G*_calc_ = *α*•(MRC-MMGBSA score)+*β*, was applied.

**Figure 4:**
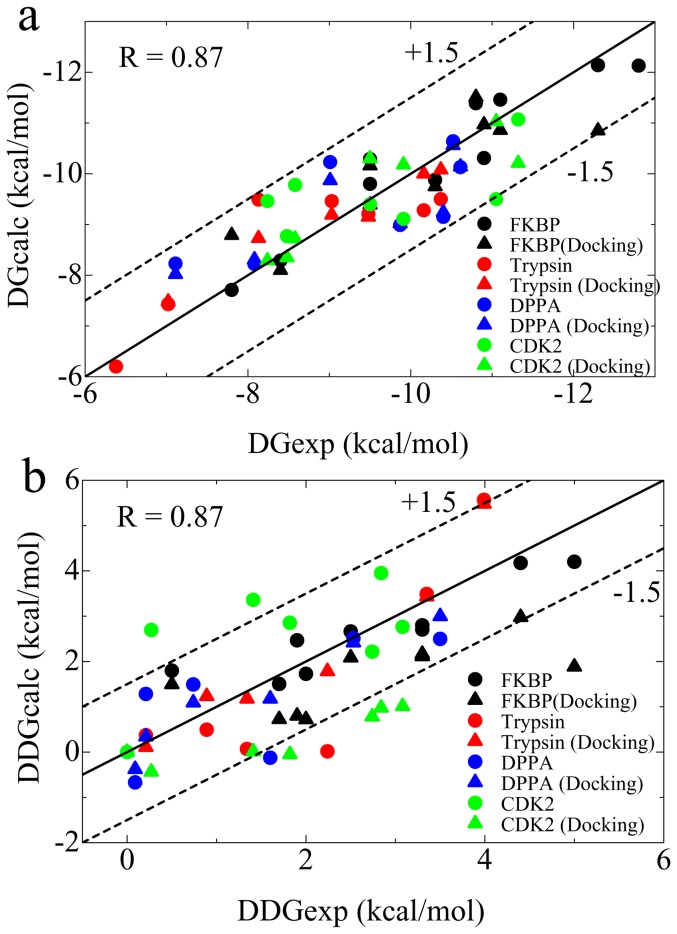
Correlation between calculated and experimental Δ*G* (a) and ΔΔ*G* (b). The correlation coefficients (*R* values) are shown in the figure. The spheres are the results obtained from X-ray, NMR or model structures, and the triangles are the results obtained from top-scored docking structures.

With *ε* = 4, we obtained a value of {*α*, *β*} = {0.25, 0.91} for the FKBP system, {0.12, −2.83} for the trypsin, {0.15, 2.27} for the DPPA, and {0.38, 6.64} for the CDK2 system, by the least-square fitting method. The Δ*G*_calc_ results were in excellent agreement with the experimental Δ*G* for all systems in which the Δ*G* error was less than ±1.5 kcal•mol^−1^ (Figure 4A). The weighting factor *α* showed similar values for all systems (*α*≈0.20), while the *y* intercept *β* varied within the range of −2.83 to +6.64. These results indicate clearly that the MRC-MMGBSA score bears a linear relation to the experimental Δ*G* with a generalized weighting factor of *α* = 0.20; however, the *y* intercept (the baseline) is system dependent. Therefore, we cannot determine a generalized value of *β*, which could be applied to all arbitrary target proteins.

Although we cannot calculate the absolute Δ*G* due to the indeterminability of the generalized *β*, the binding free energy difference (ΔΔ*G*) can be estimated from the MRC-MMGBSA score by using the generalized *α* = 0.20. To estimate the ΔΔ*G*, we used the ligand with the highest Δ*G*_exp_ as the reference for each system, ΔΔ*G*_X,system_ = Δ*G*_X,system_–Δ*G*_reference,system_ (Tables S1, S2, S3, and S4). Figure 4B shows the correlation between the calculated and experimental ΔΔ*G*. Nearly all of the ligand ΔΔ*G* errors were within ±1.5 kcal•mol^−1^ of the experimental values, although we used a variety of target proteins and ligands to assess the MRC-MMGBSA approach in this study. This result argues strongly for the use of the MRC-MMGBSA method as an efficient tool in computer-aided drug screening, because the MRC-MMGBSA scores can be directly compared with experimental binding affinities.

## Discussion

We have presented an efficient computational method for calculating ligand binding affinities, based on the MM-GBSA approach and Jarzynski identity, namely, the MRC-MMGBSA method. MRC-MMGBSA scores can correctly rank the ligands according to their binding affinities for a variety of target proteins (small-, medium-, and large-sized), and a variety of ligand sizes and net charges. Most notably, the MRC-MMGBSA method can be easily applied to flexible proteins, which undergo conformational change such as the open-close motion upon ligand binding. In addition, the optimal docking structure can be determined from among the multiple docking poses by ranking the MRC-MMGBSA scores. Importantly, the MRC-MMGBSA score shows a linear response to the experimental Δ*G*, and thus the ΔΔ*G* of the ligand binding can be estimated from the MRC-MMGBSA score simply by multiplying it by the weighting factor. The error of the ΔΔ*G* calculation is within ±1.5 kcal•mol^−1^ of the experimental ΔΔ*G*, which is sufficiently accurate to be directly compared with the experimental values.

In summary, we have proposed an effective strategy for the post-docking process in computer-aided drug screening. The first step is to determine the optimal docking structure from among the multiple docking poses, using the S-MMGBSA method. The second step is to rank these optimal docking structures according to the binding free energies obtained by S-MMGBSA analysis. After the ranking, the top several tens percent of the optimal docking structures may be selected for the next step in the analysis. The first and second steps, using the S-MMGBSA approach, provide an efficient first filter with which to narrow down the candidates, because the S-MMGBSA approach is highly efficient at determining the optimal docking structure and can roughly rank the ligands according to their binding affinities. The third step is to rank the ligands using the MRC-MMGBSA approach, and then select the top several tens of the ligands, according to their MRC-MMGBSA score (or ΔΔ*G*), for the next step. Finally, the absolute binding free energies of the several tens of selected ligands are calculated by using a more rigorous method such as PDLD/S-LRA or LIE. It is believed that this strategy would both reduce the loss of potential ligands and show the best overall performance, at present time, in term of computational costs and accuracy.

## Methods

### Calculation of the MRC-MMGBSA score

Jarzynski identity is an equation in statistical mechanics that relates free energy differences between two equilibrium states and non-equilibrium processes [27], [28]. 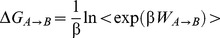
(1)


In principle, the work required for ligand binding, *W*_bound→unbound_, can be measured by single-molecule experimentation using, for example, atomic force microscopy or laser optical tweezers [43]. Computationally, it can be estimated by single-molecule pulling simulations such as steered MD simulations [16], [29]–[31]. In the quasi-static process wherein the pulling is done infinitely slowly, at the zero time limit, an exponential average of the work from state *i* to *i*+1 (*W_i_*) corresponds to that of the potential energy difference between state *i* and *i*+1 (*U_i_*_+1_−*U_i_*). Thus, the Jarzynski identity, Equation (1), becomes
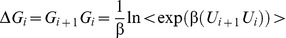
(2)


Because of the difficulty involved in estimating the exponential average, and since the exponential average strongly depends on the tails of the work distribution, it is very difficult to calculate accurately the free energy by Equation (2). In practice, a number of trajectories are generated by repeating the steered MD simulations, varying conditions such as the initial structures, pulling speed, and/or pulling directions [16], [29], [30]. However, such steered MD simulations are too expensive to employ in computer-aided drug screening using a large chemical compound library. In this study, then, as an alternative to steered MD simulations, multiple conformations, varying the protein-ligand distance *r* and orientation of ligands, are randomly generated (Figure 1). Here, we introduced an approximation that randomly generates the trajectories in order to conserve computational resources, and we calculated energies using energy-minimized but not MD structures (excluding thermal fluctuation). In addition, we also employed the implicit solvent model by using the MM-GBSA method to calculate the solvation energies. Thus, the calculated Δ*G* using Equation (2) is no longer the absolute Δ*G*, and we define the calculated Δ*G* as the ‘score’:
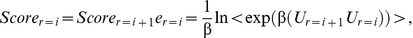
(3)


and
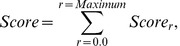
(4)where *r* is the protein-ligand distance. Because the score is calculated on the basis of the MM-GBSA calculation by using the multiple random conformations, we call the score an ‘MRC-MMGBSA score’.

### Protocol for Calculating the Energy

We evaluated the MRC-MMGBSA scores for four target proteins: FKBP, trypsin, DPPA, and CDK2. The X-ray, NMR or modeled structures of the respective protein-ligand complexes were used as the template conformations (Tables S1, S2, S3, and S4). The ff03 force field [44] was adopted for the receptor proteins. For the ligands, the parameters were determined using the *Antechamber* module (version 1.27), utilizing the general atom force field (GAFF) [45]. Partial charges for the ligands, not included in the standard ff03 parameter set, were calculated at the RHF/6-31G*/B3LYP/cc-pVTZ SCRF level with Gaussian03 and the restrained electrostatic potential (RESP) method [46].

The energy of the template conformations was minimized until the RMS of the Cartesian elements of the gradient was less than 0.1 kcal•mol^−1^ in the Generalized Born/surface area (GBSA) implicit solvent model (the method for the minimization was switched from steepest descent to conjugate gradient after 100 step), and then 10 ps-MD simulations using the GBSA method were performed in order to sample the multiple protein-ligand conformations of the ligand-bound state (protein-ligand distance *r* = 0.0). During the MD simulations, the temperature was kept constant at 300 K by a Langevin thermostat with a collision frequency γ of 2.0 ps. A time step of 1.0 fs was used. All bond lengths involving hydrogen atoms were constrained to the equilibrium length by using the SHAKE method [47]. All energy minimizations and MD simulations were performed using Amber 11 [48].

The MD trajectories were collated with a saved snapshot every 0.1 ps, in order to sample a hundred conformations of the protein-ligand distance *r* = 0.0. For the other protein-ligand distance *r* values, a hundred conformations were randomly generated for each protein-ligand distance *r*, based on the samples at *r* = 0.0. In randomly generating the multiple protein-ligand conformations, we set *r* to 0.5, 1.0, 2.0, 3.0 …. 10.0, and the ligand molecule was allowed to randomly rotate without internal conformational change. In this process, six random numbers were generated, *x*, *y*, and *z* of rotation angles (*θ_x_*, *θ_y_*, *θ_z_*) and protein-ligand distance *r* (*r_x_*, *r_y_*, and *r_z_*) [wherein *r* ^2^ = (*r_x_* ^2^+*r_y_* ^2^+*r_z_* ^2^)]. Figure 1 shows a schematic representation of the method for generating the multiple random conformations. Because we used twelve points for *r*, a total of 1,200 conformations were randomly generated. The multiple conformations randomly generated were subjected to energy minimization in the implicit salvation model (GBSA method) (the method for the minimization was switched from steepest descent to conjugate gradient after 10 step). The number of steps for the energy minimization, *N*min, was set at 100. The potential energies at the final energy minimization step were sampled in order to calculate the score.

## Supporting Information

Figure S1 **Structure of ligands for the FKBP system.**
(TIF)

Figure S2 **Structure of ligands for the trypsin system.**
(TIF)

Figure S3 **Structure of ligands for the DPPA system.**
(TIF)

Figure S4 **Structure of ligands for the CDK2 system.**
(TIF)

Table S1 **PDB code, experimental Δ** ***G*** **, net charge, and reference of ligands for the FKBP system.**
(DOC)

Table S2 **PDB code, experimental Δ** ***G*** **, net charge, and reference of ligands for the trypsin system.**
(DOC)

Table S3 **PDB code, experimental Δ** ***G*** **, net charge, and reference of ligands for the DPPA system.**
(DOC)

Table S4 **PDB code, experimentalΔ** ***G*** **, net charge, and reference of ligands for the CDK2 system.**
(DOC)

Table S5 **Heavy atom RMSD of ligands with the top-scored docking poses, obtained by the MRC-MMGBSA, S-MMGBSA, and docking procedures, for the FKBP system.**
(DOC)

Table S6 **Heavy atom RMSD of ligands with the top-scored docking poses, obtained by the MRC-MMGBSA, S-MMGBSA, and docking procedures, for the trypsin system.**
(DOC)

Table S7 **Heavy atom RMSD of ligands with the top-scored docking poses, obtained by the MRC-MMGBSA, S-MMGBSA, and docking procedures, for the DPPA system.**
(DOC)

Table S8 **Heavy atom RMSD of ligands with the top-scored docking poses, obtained by the MRC-MMGBSA, S-MMGBSA, and docking procedures, for the CDK2 system.**
(DOC)
