# Supplementary figures and images for: An Efficient Computational Method for Calculating Ligand Binding Affinities
Source: PLoS One. 2012 Aug 20;7(8):e42846. doi: 10.1371/journal.pone.0042846 (PMC3423425; doi:10.1371/journal.pone.0042846)

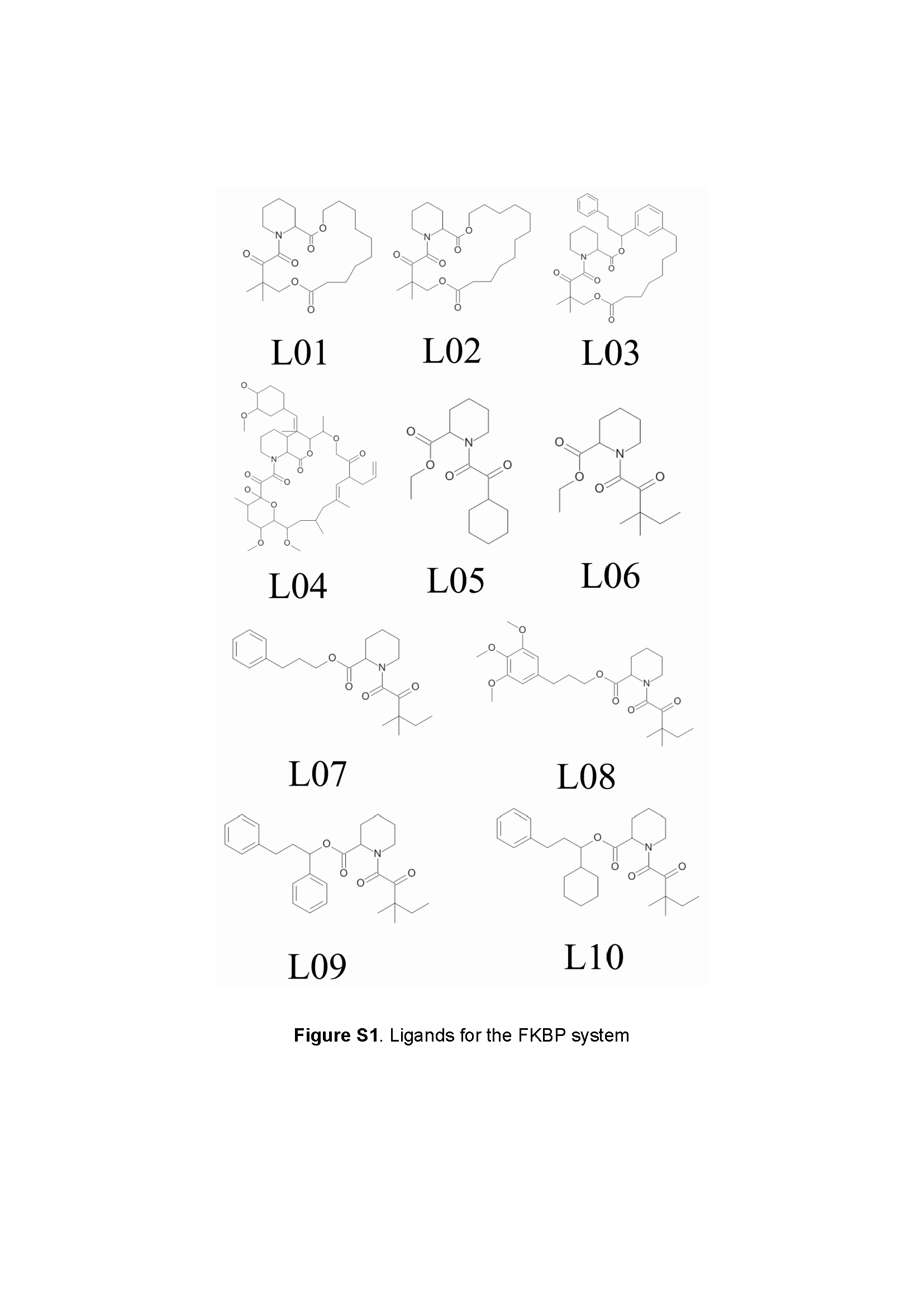

Supplement: Figure S1 — Structure of ligands for the FKBP system. (TIF) [file pone.0042846.s001.tif]

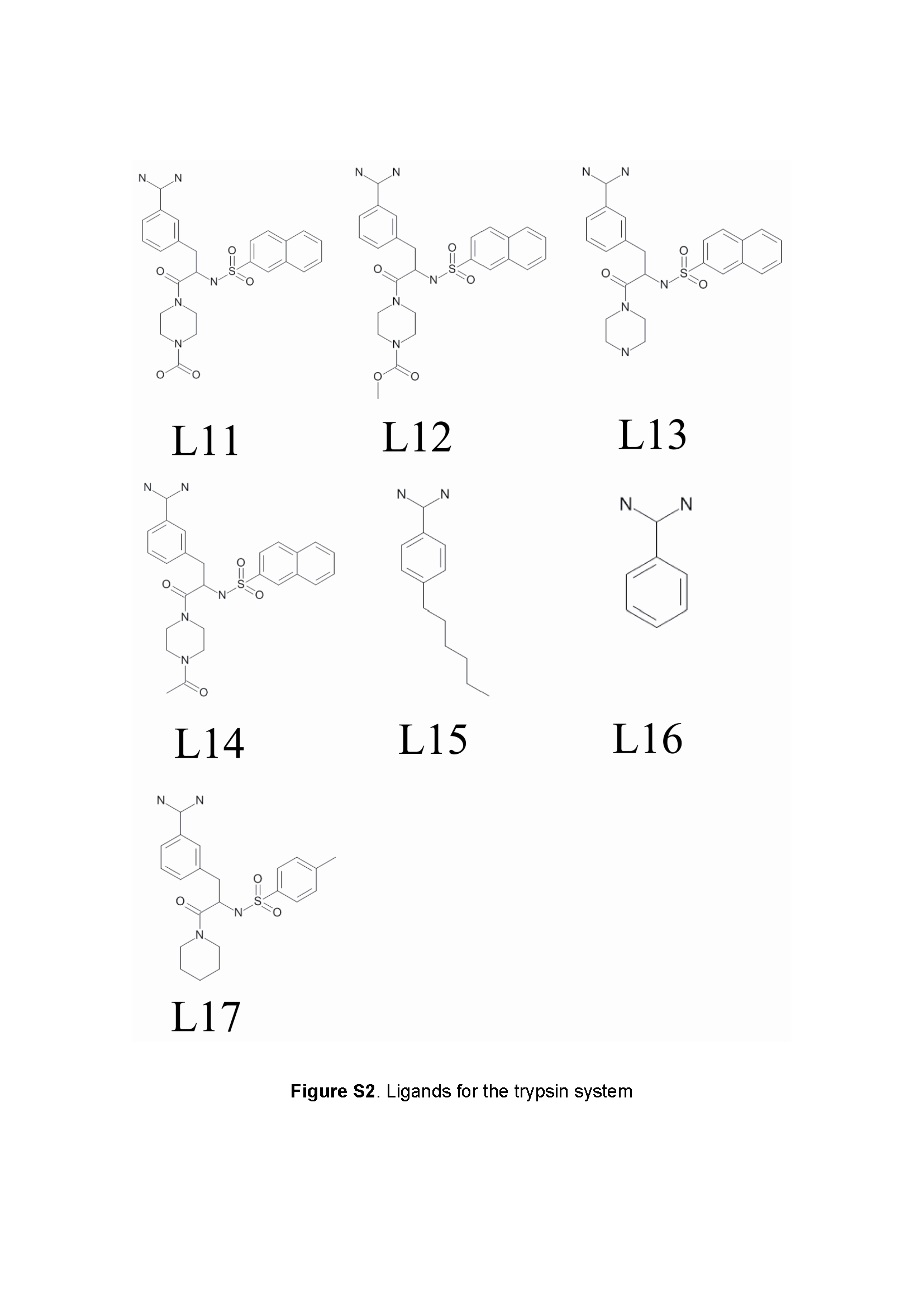

Supplement: Figure S2 — Structure of ligands for the trypsin system. (TIF) [file pone.0042846.s002.tif]

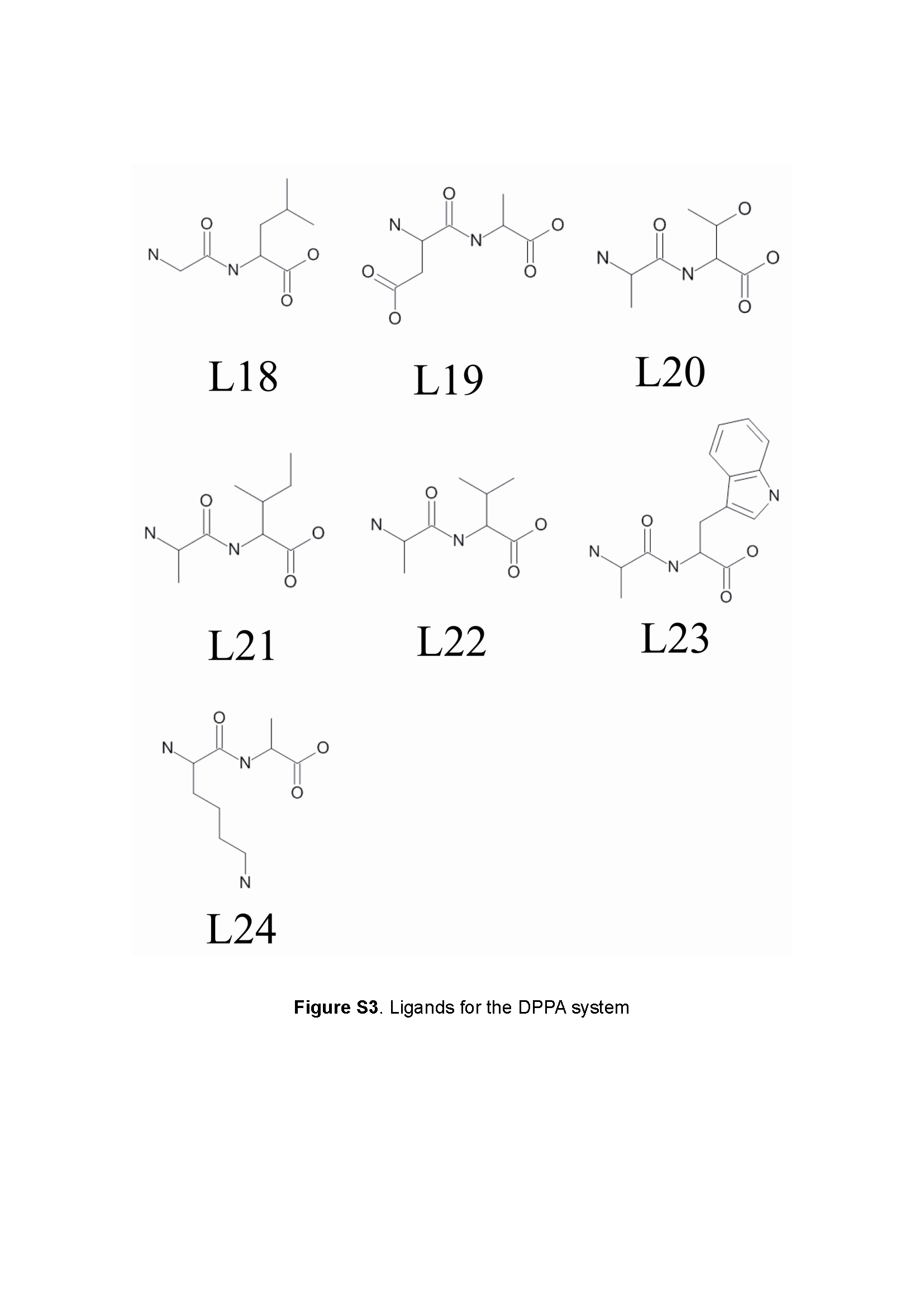

Supplement: Figure S3 — Structure of ligands for the DPPA system. (TIF) [file pone.0042846.s003.tif]

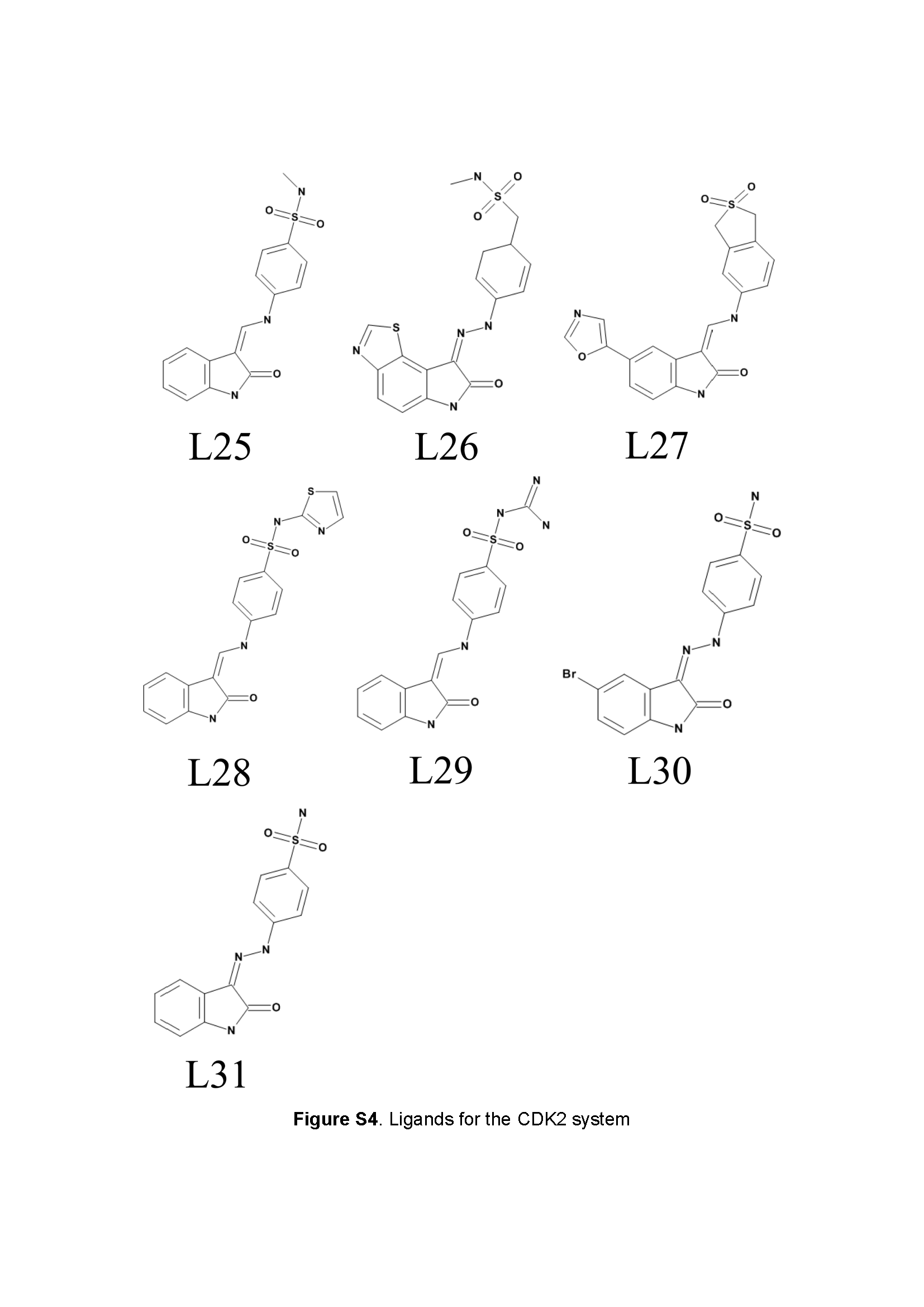

Supplement: Figure S4 — Structure of ligands for the CDK2 system. (TIF) [file pone.0042846.s004.tif]
