# Supplementary material for: An Efficient Computational Method for Calculating Ligand Binding Affinities
Source: PLoS One. 2012 Aug 20;7(8):e42846. doi: 10.1371/journal.pone.0042846 (PMC3423425; doi:10.1371/journal.pone.0042846)
Supplement: Table S1 — PDB code, experimental Δ G , net charge, and reference of ligands for the FKBP system. (DOC) [file pone.0042846.s005.doc]

**Table S1**. Ligands for the FKBP system

| Ligand | PDB code | Δ*G*_exp_ (kcal∙mol^−1^) | Net charge | Ref. |
| --- | --- | --- | --- | --- |
| L01 | Model^†^ | −11.1 | 0.0 | 1 |
| L02 | 1fki | −9.5 | 0.0 | 1 |
| L03 | Model | −12.3 | 0.0 | 1 |
| L04 | 1fkf | *−12.8 | 0.0 | 1 |
| L05 | Model | −7.8 | 0.0 | 1 |
| L06 | Model | −8.4 | 0.0 | 1 |
| L07 | Model | −9.5 | 0.0 | 1 |
| L08 | Model | −10.8 | 0.0 | 1 |
| L09 | 1fkg | −10.9 | 0.0 | 1 |
| L10 | 1fkh | −11.1 | 0.0 | 1 |

*The reference Δ*G* for calculating ΔΔ*G*_FKBP_.

^†^The model structure is built by fitting together a pipecolate and an *α*–keto amide region (see Figure S1).

**Reference**

[1] Holt DA, Luengo JI, Yamashita DS, Oh HJ, Konialian AL, et al. (1993) Design, synthesis, and kinetic evaluation of high−affinity FKBP ligands and the X−ray crystal structures of their complexes with FKBP12. J Am Chem Soc 115: 9925−9938.
