# Supplementary material for: An Efficient Computational Method for Calculating Ligand Binding Affinities
Source: PLoS One. 2012 Aug 20;7(8):e42846. doi: 10.1371/journal.pone.0042846 (PMC3423425; doi:10.1371/journal.pone.0042846)
Supplement: Table S2 — PDB code, experimental Δ G , net charge, and reference of ligands for the trypsin system. (DOC) [file pone.0042846.s006.doc]

**Table S2**. Ligands for the trypsin system

| Ligand | PDB code | Δ*G*_exp_ (kcal∙mol^−1^) | Net charge | Ref. |
| --- | --- | --- | --- | --- |
| L11 | 1k1i | −8.69 | 0.0 | 1 |
| L12 | 1k1j | *−10.41 | +1.0 | 1 |
| L13 | 1k1l | −9.74 | +1.0 | 1 |
| L14 | 1k1m | −10.16 | +1.0 | 1 |
| L15 | Model^†^ | −7.02 | +1.0 | 2 |
| L16 | 3ptb | −6.38 | +1.0 | 2 |
| L17 | 1pph | −8.13 | +1.0 | 3 |

*The reference Δ*G* for calculating ΔΔ*G*_trypsin_

^†^The model structure is built by fitting an amidine region (see Figure S2).

**References**

[1] Dullweber F, Stubbs MT, Musil D, Stürzebecher J, Klebe G. (2001) Factorising ligand affinity: a combined thermodynamics and crystallographic study of trypsin and thrombin inhibitor. J Mol Biol 313: 593−614.

[2] Talhout R, Villa A, Mark AE, Engberts JBFN. (2003) Understanding binding affinity: a combined isothermal titration calorimetry/molecular dynamics study of the binding of a series of hydrophobically modified benzamidinium chloride inhibitors to trypsin. J Am Chem Soc 125: 10570−10579.

[3] Turk D, Stürzebecher J, Bode W. (1991) Geometry of binding of the *N*α–tosylated piperidides of *m*–amidino–, *p*–amidino–, and *p*–guanidine phenylalanine to thrombin and trypsin: X–ray crystal structures of their trypsin complexes and modeling of their thrombin complexes. FEBS Lett 287: 133–138.
