# Supplementary material for: An Efficient Computational Method for Calculating Ligand Binding Affinities
Source: PLoS One. 2012 Aug 20;7(8):e42846. doi: 10.1371/journal.pone.0042846 (PMC3423425; doi:10.1371/journal.pone.0042846)
Supplement: Table S3 — PDB code, experimental Δ G , net charge, and reference of ligands for the DPPA system. (DOC) [file pone.0042846.s007.doc]

**Table S3**. Ligands for the DPPA system

| Ligand | PDB code | Δ*G*_exp_ (kcal∙mol^−1^) | Net charge | Ref. |
| --- | --- | --- | --- | --- |
| L18 (GL)^†^ | 1dpp | −8.08 | 0.0 | 1 |
| L19 (DA) | Model^‡^ | −7.11 | −1.0 | 1 |
| L20 (AT) | Model | −10.16 | 0.0 | 1 |
| L21 (AI) | Model | −9.87 | 0.0 | 1 |
| L22 (AV) | Model | *−10.52 | 0.0 | 1 |
| L23 (AW) | Model | −9.01 | 0.0 | 1 |
| L24 (KA) | Model | −10.40 | +1.0 | 1 |
| Apo−receptor | 1dpe |  | −8.0 | 1 |

*The reference Δ*G* for calculating ΔΔ*G*_DPPA_

^†^Amino acid sequence is indicated in the parenthesis by single−letter representation.

^‡^The model structure is built by fitting main chain atoms (see Figure S3).

**Reference**

[1] Payne JW, Grail BM, Gupta S, Ladbury JE, Marshall NJ, et al. (2000) Structural basis for recognition of dipeptides by peptide transporters. Arch Biochem Biophys 384: 9–23.
