# Supplementary material for: An Efficient Computational Method for Calculating Ligand Binding Affinities
Source: PLoS One. 2012 Aug 20;7(8):e42846. doi: 10.1371/journal.pone.0042846 (PMC3423425; doi:10.1371/journal.pone.0042846)
Supplement: Table S4 — PDB code, experimentalΔ G , net charge, and reference of ligands for the CDK2 system. (DOC) [file pone.0042846.s008.doc]

**Table S4**. Ligands for the CDK2 system

| Ligand | PDB code | Δ*G*_exp_ (kcal∙mol^−1^) | Net charge | Ref. |
| --- | --- | --- | --- | --- |
| L25 | 1ke5 | −8.58 | 0.0 | 1 |
| L26 | 1ke6 | *−11.32 | 0.0 | 1 |
| L27 | 1ke7 | −11.05 | 0.0 | 1 |
| L28 | 1ke8 | −8.24 | 0.0 | 1 |
| L29 | 1ke9 | -8.48 | 0.0 | 1 |
| L30 | 1fvt | -9.91 | 0.0 | 1 |
| L31 | Model^†^ | −9.50 | 0.0 | 1 |

*The reference Δ*G* for calculating ΔΔ*G*_CDK2_

^†^The model structure is built by replacing from Br to H in L30 (see Figure S4).

**Reference**

[1] Bramson HN, Corona J, Davis ST, Dickerson SH, Edelstein M, et al. (2001) Oxindole–based inhibitor of cyclin–dependent kinase 2 (CDK2): design, synthesis, enzymatic activities, and X–ray crystallographic analysis. J Med Chem 44: 4339–4358.
