# Supplementary material for: An Efficient Computational Method for Calculating Ligand Binding Affinities
Source: PLoS One. 2012 Aug 20;7(8):e42846. doi: 10.1371/journal.pone.0042846 (PMC3423425; doi:10.1371/journal.pone.0042846)
Supplement: Table S5 — Heavy atom RMSD of ligands with the top-scored docking poses, obtained by the MRC-MMGBSA, S-MMGBSA, and docking procedures, for the FKBP system. (DOC) [file pone.0042846.s009.doc]

**Table S5**. Heavy atom RMSD of ligands with the top–scored docking poses, obtained by the MRC–MMGBSA, S–MMGBSA, and docking procedures, for the FKBP system

|  | L01* | L02 | L03 | L04 | L05 | L06 | L07 | L08 | L09 | L10 |
| --- | --- | --- | --- | --- | --- | --- | --- | --- | --- | --- |
| MRC–MMGBSA^†^ |  |  |  |  |  |  |  |  |  |  |
| ε=1 | 0.32^¶^ | 1.35^¶^ | 1.12^¶^ | 1.99 | 0.73^¶^ | 6.14 | 1.71^¶^ | 2.54 | 1.88^¶^ | 1.45^¶^ |
| ε=2 | 0.32^¶^ | 1.35^¶^ | 1.12^¶^ | 1.89 | 0.73^¶^ | 6.14 | 1.71^¶^ | 2.54 | 1.88^¶^ | 1.45^¶^ |
| ε=4 | 0.32^¶^ | 1.35^¶^ | 1.12^¶^ | 1.18^¶^ | 0.73^¶^ | 1.31^¶^ | 1.71^¶^ | 1.76^¶^ | 1.88^¶^ | 1.45^¶^ |
| S–MMGBSA^‡^ |  |  |  |  |  |  |  |  |  |  |
| ε=1 | 0.66^¶^ | 1.62^¶^ | 1.39^¶^ | 1.86^¶^ | 0.94^¶^ | 6.12 | 1.52^¶^ | 2.35 | 1.80^¶^ | 1.39^¶^ |
| ε=2 | 0.66^¶^ | 1.62^¶^ | 1.39^¶^ | 1.86^¶^ | 0.94^¶^ | 0.85^¶^ | 1.52^¶^ | 2.35 | 1.80^¶^ | 1.39^¶^ |
| ε=4 | 0.66^¶^ | 1.62^¶^ | 1.39^¶^ | 1.86^¶^ | 0.94^¶^ | 0.85^¶^ | 1.52^¶^ | 2.35 | 1.80^¶^ | 1.39^¶^ |
| Autodock^§^ |  |  |  |  |  |  |  |  |  |  |
|  | 0.85^¶^ | 7.47 | 1.50^¶^ | 1.07^¶^ | 1.01^¶^ | 0.75^¶^ | 7.85 | 1.82^¶^ | 6.56 | 6.60 |

*All ligand structures are shown in Figure S1.

^†^RMSD of ligand between reference structure and docking pose after structural relaxation by MD simulation.

^‡^RMSD of ligand between reference structure and docking pose after structural optimization by energy minimization.

^§^RMSD of ligand between reference structure and docking pose obtained by AutoDock Vina.

^¶^Docking pose showing the lowest RMSD among the five docking poses.
