# Supplementary material for: An Efficient Computational Method for Calculating Ligand Binding Affinities
Source: PLoS One. 2012 Aug 20;7(8):e42846. doi: 10.1371/journal.pone.0042846 (PMC3423425; doi:10.1371/journal.pone.0042846)
Supplement: Table S6 — Heavy atom RMSD of ligands with the top-scored docking poses, obtained by the MRC-MMGBSA, S-MMGBSA, and docking procedures, for the trypsin system. (DOC) [file pone.0042846.s010.doc]

**Table S6**. Heavy atom RMSD of ligands with the top–scored docking poses, obtained by the MRC–MMGBSA, S–MMGBSA, and docking procedures, for the trypsin system

|  | L11* | L12 | L13 | L14 | L15 | L16 | L17 |
| --- | --- | --- | --- | --- | --- | --- | --- |
| MRC–MMGBSA^†^ |  |  |  |  |  |  |  |
| ε=1 | 1.98^¶^ | 1.49^¶^ | 1.70^¶^ | 2.33 | 1.22 | 1.96 | 2.62^¶^ |
| ε=2 | 2.50 | 1.49^¶^ | 1.70^¶^ | 2.33 | 1.22 | 1.12^¶^ | 2.62^¶^ |
| ε=4 | 2.50 | 1.49^¶^ | 1.70^¶^ | 2.33 | 1.22 | 1.12^¶^ | 2.62^¶^ |
| S–MMGBSA^‡^ |  |  |  |  |  |  |  |
| ε=1 | 1.99^¶^ | 1.27 | 1.37^¶^ | 1.17^¶^ | 1.04^¶^ | 1.08^¶^ | 3.64 |
| ε=2 | 1.99^¶^ | 1.21^¶^ | 1.37^¶^ | 1.17^¶^ | 1.04^¶^ | 1.08^¶^ | 3.64 |
| ε=4 | 2.03 | 1.21^¶^ | 1.37^¶^ | 1.17^¶^ | 1.04^¶^ | 1.93 | 3.65 |
| Autodock^§^ |  |  |  |  |  |  |  |
|  | 4.94 | 1.22^¶^ | 2.57 | 4.01 | 1.08 | 1.14^¶^ | 3.29 |

*All ligand structures are shown in Figure S2.

^†^RMSD of ligand between reference structure and docking pose after structural relaxation by MD simulation.

^‡^RMSD of ligand between reference structure and docking pose after structural optimization by energy minimization.

^§^RMSD of ligand between reference structure and docking pose obtained by AutoDock Vina

^¶^Docking pose showing the lowest RMSD among the five docking poses.

^║^All RMSD values were calculated using only amidine group of ligands, because the amidine group is the common binding element for all ligands used in this study, and interacts with the receptor by forming hydrogen bonds. The other groups of ligands are extruded from the binding pocket and are thereby relatively flexible.
