# Supplementary material for: An Efficient Computational Method for Calculating Ligand Binding Affinities
Source: PLoS One. 2012 Aug 20;7(8):e42846. doi: 10.1371/journal.pone.0042846 (PMC3423425; doi:10.1371/journal.pone.0042846)
Supplement: Table S7 — Heavy atom RMSD of ligands with the top-scored docking poses, obtained by the MRC-MMGBSA, S-MMGBSA, and docking procedures, for the DPPA system. (DOC) [file pone.0042846.s011.doc]

**Table S7**. Heavy atom RMSD of ligands with the top–scored docking poses, obtained by the MRC–MMGBSA, S–MMGBSA, and docking procedures, for the DPPA system

|  | L18* | L19 | L20 | L21 | L22 | L23 | L24 |
| --- | --- | --- | --- | --- | --- | --- | --- |
| MRC–MMGBSA^†^ |  |  |  |  |  |  |  |
| ε=1 | 1.33^¶^ | 2.23^¶^ | 1.82^¶^ | 1.29^¶^ | 1.50^¶^ | 3.31^¶^ | 1.83^¶^ |
| ε=2 | 1.33^¶^ | 2.23^¶^ | 1.82^¶^ | 1.29^¶^ | 1.50^¶^ | 3.31^¶^ | 1.83^¶^ |
| ε=4 | 1.33^¶^ | 2.23^¶^ | 1.82^¶^ | 1.29^¶^ | 1.50^¶^ | 3.31^¶^ | 1.83^¶^ |
| S–MMGBSA^‡^ |  |  |  |  |  |  |  |
| ε=1 | 1.03^¶^ | 1.80^¶^ | 1.37^¶^ | 1.63^¶^ | 1.47^¶^ | 3.32^¶^ | 2.00^¶^ |
| ε=2 | 1.03^¶^ | 1.80^¶^ | 1.37^¶^ | 1.63^¶^ | 1.47^¶^ | 3.32^¶^ | 2.00^¶^ |
| ε=4 | 1.03^¶^ | 1.80^¶^ | 1.37^¶^ | 1.63^¶^ | 1.47^¶^ | 3.32^¶^ | 2.00^¶^ |
| Autodock^§^ |  |  |  |  |  |  |  |
|  | 1.06^¶^ | 1.84^¶^ | 1.40^¶^ | 1.67^¶^ | 1.50^¶^ | 6.32 | 2.07 |

*All ligand structures are shown in Figure S3.

^†^RMSD of ligand between reference structure and docking pose after structural relaxation by MD simulation.

^‡^RMSD of ligand between reference structure and docking pose after structural optimization by energy minimization.

^§^RMSD of ligand between reference structure and docking pose obtained by AutoDock Vina

^¶^Docking pose showing the lowest RMSD among the five docking poses.
