# Supplementary material for: An Efficient Computational Method for Calculating Ligand Binding Affinities
Source: PLoS One. 2012 Aug 20;7(8):e42846. doi: 10.1371/journal.pone.0042846 (PMC3423425; doi:10.1371/journal.pone.0042846)
Supplement: Table S8 — Heavy atom RMSD of ligands with the top-scored docking poses, obtained by the MRC-MMGBSA, S-MMGBSA, and docking procedures, for the CDK2 system. (DOC) [file pone.0042846.s012.doc]

**Table S8**. Heavy atom RMSD of ligands with the top–scored docking poses, obtained by the MRC–MMGBSA, S–MMGBSA, and docking procedures, for the CDK2 system

|  | L25 | L26* | L27 | L28 | L29 | L30 | L31 |
| --- | --- | --- | --- | --- | --- | --- | --- |
| MRC–MMGBSA^†^ |  |  |  |  |  |  |  |
| ε=1 | 1.27^¶^ | 8.02 | 5.25 | 1.04^¶^ | 1.70^¶^ | 1.57^¶^ | 2.07^¶^ |
| ε=2 | 1.27^¶^ | 8.02 | 2.33 | 1.04^¶^ | 2.94 | 1.57^¶^ | 2.07^¶^ |
| ε=4 | 1.27^¶^ | 2.48^¶^ | 2.31^¶^ | 1.04^¶^ | 2.14 | 1.57^¶^ | 2.07^¶^ |
| S–MMGBSA^‡^ |  |  |  |  |  |  |  |
| ε=1 | 0.30^¶^ | 3.76 | 2.27^¶^ | 8.95 | 2.38 | 2.09^¶^ | 3.41 |
| ε=2 | 0.30^¶^ | 3.76 | 2.27^¶^ | 8.95 | 1.29^¶^ | 2.09^¶^ | 0.64^¶^ |
| ε=4 | 0.30^¶^ | 3.76 | 2.27^¶^ | 8.95 | 1.29^¶^ | 3.63 | 0.64^¶^ |
| Autodock^§^ |  |  |  |  |  |  |  |
|  | 0.38^¶^ | 1.41^¶^ | 2.28^¶^ | 2.35 | 2.38 | 2.38^¶^ | 2.72 |

*All ligand structures are shown in Figure S4.

^†^RMSD of ligand between reference structure and docking pose after structural relaxation by MD simulation.

^‡^RMSD of ligand between reference structure and docking pose after structural optimization by energy minimization.

^§^RMSD of ligand between reference structure and docking pose obtained by AutoDock Vina

^¶^Docking pose showing the lowest RMSD among the five docking poses.
